# Short stature as a presenting symptom of attenuated Mucopolysaccharidosis type I: case report and clinical insights

**DOI:** 10.1186/s12902-018-0311-x

**Published:** 2018-11-12

**Authors:** Ana Maria Martins, Kristin Lindstrom, Sandra Obikawa Kyosen, Maria Veronica Munoz-Rojas, Nathan Thibault, Lynda E. Polgreen

**Affiliations:** 1 Universidade Federal de São Paulo, São Paulo, Brazil; 2 Phoenix Children’s Hospital, Phoenix, AZ USA; 3 Sanofi Genzyme, Cambridge, MA USA; 4 Los Angeles Biomedical Research Institute at Harbor-UCLA Medical Center, 1124 West Carson Street, Liu Research Building, Torrance, CA 90502 USA

**Keywords:** MPS I diagnosis, MPS I signs and symptoms, Growth delay, Physician awareness, Early diagnosis, Short stature

## Abstract

**Background:**

Mucopolysaccharidosis type I (MPS I) results in significant disease burden and early treatment is important for optimal outcomes. Recognition of short stature and growth failure as symptoms of MPS I among pediatric endocrinologists may lead to earlier diagnosis and treatment.

**Case presentation:**

A male patient first began experiencing hip pain at 5 years of age and was referred to an endocrinologist for short stature at age 7. Clinical history included recurrent respiratory infections, sleep apnea, moderate joint contractures, mild facial dysmorphic features, scoliosis, and umbilical hernia. Height was more than − 2 SD below the median at all time points. Growth velocity was below the 3rd percentile. Treatment for short stature included leuprolide acetate and recombinant human growth hormone. The patient was diagnosed with MPS I and began enzyme replacement therapy with laronidase at age 18.

**Conclusions:**

The case study patient had many symptoms of MPS I yet remained undiagnosed for 11 years after presenting with short stature. The appropriate path to MPS I diagnosis when patients present with short stature and/or growth failure plus one or more of the common signs of attenuated disease is described. Improved awareness regarding association of short stature and growth failure with attenuated MPS I is needed since early identification and treatment significantly decreases disease burden.

## Background

Mucopolysaccharidosis type I (MPS I) is a life-threatening, autosomal recessive disease caused by deficiency of α-L-iduronidase (IDUA), a lysosomal enzyme responsible for metabolizing the glycosaminoglycans (GAGs) dermatan and heparan sulfate [1]. MPS I has an estimated incidence of 1/100,000 live births with a spectrum of phenotypes that range from severe (Hurler syndrome) to attenuated (Hurler-Scheie and Scheie syndromes) disease, depending on neurocognitive involvement and the rate of disease progression [2–4].

MPS I results in significant disease burden, disability, and premature death from respiratory and cardiac disease if left untreated, as well as neurodegeneration in the severe phenotype [2]. Treatment options include enzyme replacement with laronidase (recombinant human IDUA; Aldurazyme®) for patients with attenuated MPS I [5–8] and hematopoietic stem cell transplantation (HSCT), with or without laronidase, for patients with severe MPS I [9–13]. Treatment outcomes depend on phenotype and age at initiation of treatment [6, 14, 15]. Early treatment, prior to irreversible damage, can delay, stabilize, or prevent disease, and is associated with substantially improved patient outcomes [5, 6, 10, 16–22]. Unfortunately, there may be considerable delays in the diagnosis of MPS I, especially for patients with attenuated disease [16, 23–25]. In a study of the diagnostic history of MPS I patients, approximately 20% of a population of 60 patients with attenuated MPS I had delays of 5 years or longer in diagnosis, and consulted between 4 and 5 specialists before receiving an MPS I diagnosis [25]. Similarly, for 18 patients with MPS I (13 of whom had attenuated disease) whose symptoms were noted at 18 months, the mean age at biochemical diagnosis was 75 months [26]. While pilot programs for MPS I NBS are in progress around the world [27], they are not universally available, and diagnostic delay persists. There has been no significant improvement in reducing the delay in diagnosis of MPS I as of 2017 [28]. Thus, it remains likely that children with undiagnosed MPS I will be referred to specialists, including endocrinologists, for their care, and awareness of the early clinical signs and symptoms remains important.

Short stature and skeletal sequelae known as dysostosis multiplex are key features among patients with MPS I [15, 24, 29–31], but MPS may be under recognized in patients presenting with short stature, particularly in patients with attenuated disease. Regardless of age and gender, children with MPS have severely disordered growth with percentile values for both longitudinal and transversal parameters (e.g., body length, trunk length, lower extremity length, shoulder breadth, and hip breadth) much lower than reference chart norms [32, 33].

Children with short stature (height less than 2 standard deviations below the mean, i.e., near the third percentile) and/or growth failure (growth rate below age-appropriate growth velocity) are often referred to pediatric endocrinologists [34, 35]. While there are multiple monogenic causes of short stature [35], attenuated MPS I should be considered in the differential diagnosis of patients with growth retardation in conjunction with any of the signs and symptoms of MPS I (Table 1), joint contractures, (particularly of hands, with claw hand deformity, although other joints can be affected), carpal tunnel syndrome (with trigger digits), umbilical and inguinal hernias, corneal clouding, hepatosplenomegaly, frequent respiratory infections, sleep apnea, cardiac valve abnormalities, and extensive surgical history. The constellation of radiologic abnormalities of ribs, extremities and spine known as dysostosis multiplex are more pronounced in severe MPS I but can develop in patients with attenuated disease at a later age. However, even patients with severe MPS I are still misdiagnosed and treated for other disorders, such as rickets [36], while patients with attenuated disease can go undiagnosed or misdiagnosed [37, 38].

**Table 1:** Common presenting/early symptoms in patients with attenuated MPS I [24, 51]

• Growth delay (normal birth weight, but growth failure or short stature) • Joint contractures (primarily in hands/claw hand deformity), joint pain and stiffness, restricted mobility • Carpal tunnel syndrome (trigger digits) • Recurrent hernias (umbilical and/or inguinal) • Corneal clouding • Hepatosplenomegaly • Skeletal abnormalities/dysostosis multiplex (e.g., kyphosis, scoliosis, hip dysplasia, flattened vertebral bodies, oar-shaped ribs, short thickened clavicles, bullet-shaped phalanges, dysplastic femoral heads, flattened acetabula, coxa valga and genu valgum deformities) • Ear/nose/throat symptoms (recurrent ear infections, noisy breathing, sleep apnea, enlarged tongue, hearing loss) • Heart murmur (valve abnormalities) • Surgical history of multiple hernia repairs, PE tubes, tonsillectomy, adenoidectomy	

All symptoms may not be present in the same patient, but are usually progressive. See Fig. 2 for the path to diagnosis

A real-world case report is presented to highlight the existence of undiagnosed MPS I patients in pediatric endocrinology clinics, and describe the appropriate path to MPS I diagnosis when patients present with short stature and/or growth failure plus one or more of the common signs of attenuated disease as described in Table 1 and Fig. 3.

## Case presentation

**Fig. 1:**
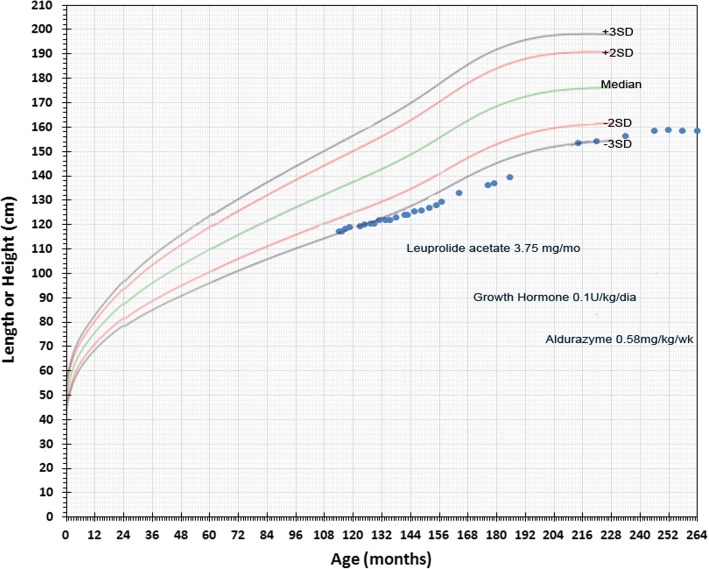
Longitudinal Growth for Patient with Attenuated MPS I from Case Study. Height of case study patient by age is shown by the blue markers with timing and duration of leuprolide acetate, growth hormone and laronidase treatments indicated. WHO Child Growth Standards are indicated

**Fig. 2:**
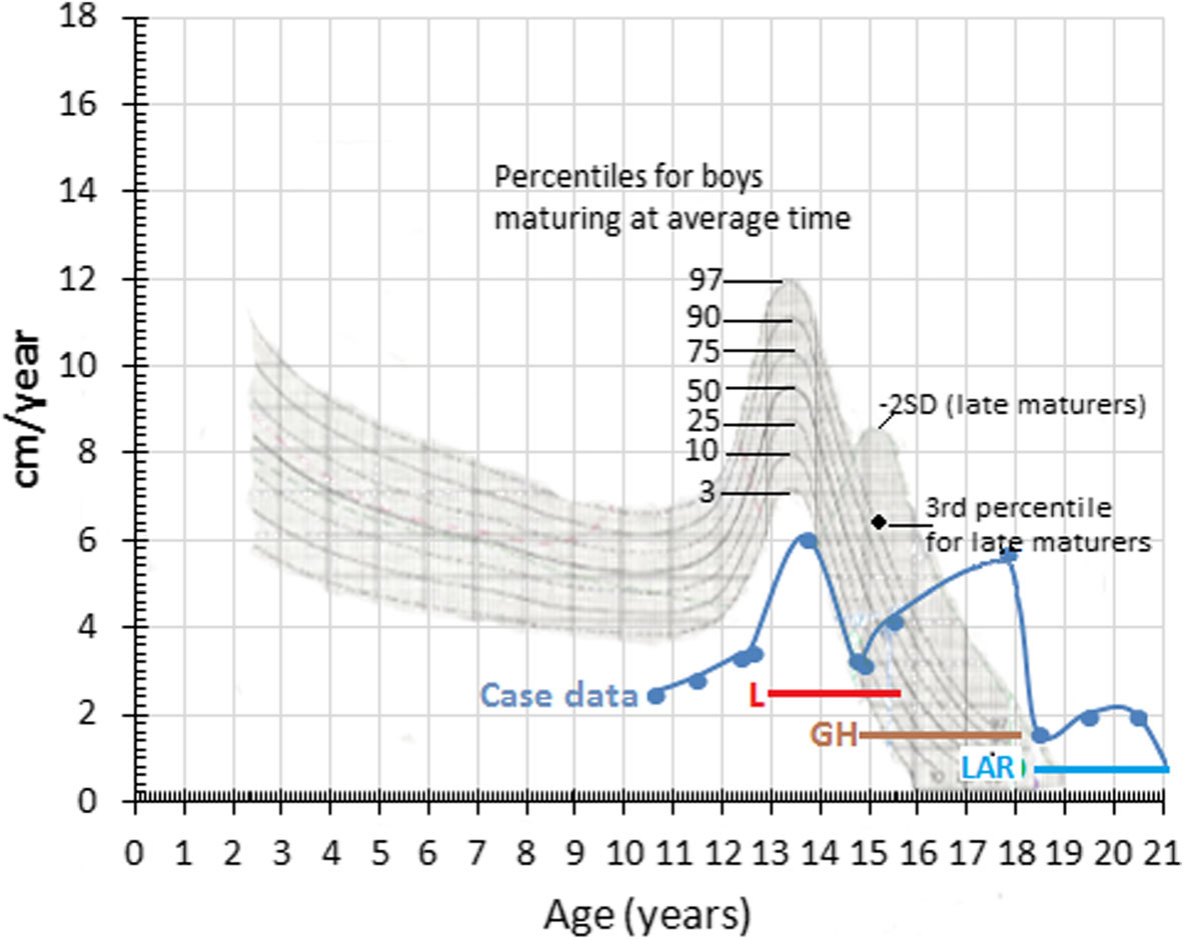
Growth Velocity of Patient with Attenuated MPS I from Case Study Relative to Growth Standards. Growth velocity by age is shown by the blue markers with timing and duration of leuprolide acetate (L), growth hormone (GH) and laronidase (LAR) treatments indicated. Treatment doses are the same as shown in Fig. 1. Percentiles for boys maturing at average time or for late maturers are shown as adapted from Tanner JM and Davies PS. Clinical longitudinal standards for height and height velocity for North American children [50]

A male patient first began experiencing hip pain at 5 years of age and was diagnosed with bilateral Legg-Calve-Perthes disease by an orthopedic surgeon (see Table 2 Timeline). The patient was not tested for juvenile idiopathic arthritis. The patient experienced recurrent upper airway tract infections during childhood and was hospitalized multiple times between 5 and 7 years of age for wheezing. He was first seen by a pediatric endocrinologist at 7 years of age due to his short stature, was treated with leuprolide acetate (3.75 mg/month) between the ages of 13 and 16 years, and recombinant human growth hormone (0.1UI/kg/day), although the patient was not labeled as growth hormone deficient (GHD), between 14 and 18 years of age. Longitudinal growth curves and growth velocity for the patient between the ages of 10 and 21 are shown in Figs. 1 and 2. Height was more than − 2 SD below the median at all time points. Growth velocity was below the 3rd percentile prior to starting growth hormone. Final height of 159 cm was reached at 20 years of age.

**Table 2:** Timeline of assessments, diagnoses and treatment

Patient age	Symptom(s)	Assessments/diagnoses	Treatment(s)
5–7 years	Hip pain, recurrent respiratory infections	Orthopedist assessment and diagnosis of bilateral Legg-Calve-Perthes disease	unknown
7–18 years	Short stature: see Figs. 1 and 2	Pediatric endocrinologist assessment	Leuprolide acetate (3.75 mg/month) ages 13–16 Growth hormone (0.1UI/kg/day) ages 14–18
18 years	Short stature, moderate joint contractures, mild facial dysmorphic features (coarsening of features), scoliosis, and an umbilical hernia	Referred to metabolic disease center by treating pulmonologist. enzyme activity screening for MPS I positive; genetic analysis positive for MPS I	Enzyme replacement therapy with laronidase (weekly 0.58 mg/kg infusions) initiated

The patient experienced sleep apnea throughout his teenage years, and at 17 years 9 months of age was referred to a metabolic disease center by a pneumologist (pulmonologist) who had seen the patient and suspected MPS. Upon examination, the patient was found to have moderate joint contractures, mild facial dysmorphic features (coarsening of features), scoliosis, and an umbilical hernia. His height at examination was 154 cm, with a z-score of − 2.94. Weight was 43.7 kg, and head circumference was 56.5 cm. Alpha-iduronidase activity on dried blood spot testing was 0.62 μmol/L/h (normal reference range 2.5–16.7), and genotype analysis revealed 2 pathogenic *IDUA* missense variants, c.1148G > A (p.R383H) and c.1598C > G (p.P533R), confirming the diagnosis of MPS I. The patient began ERT with laronidase (0.58 mg/kg/week) at age 18.

## Discussion and conclusions

**Fig. 3:**
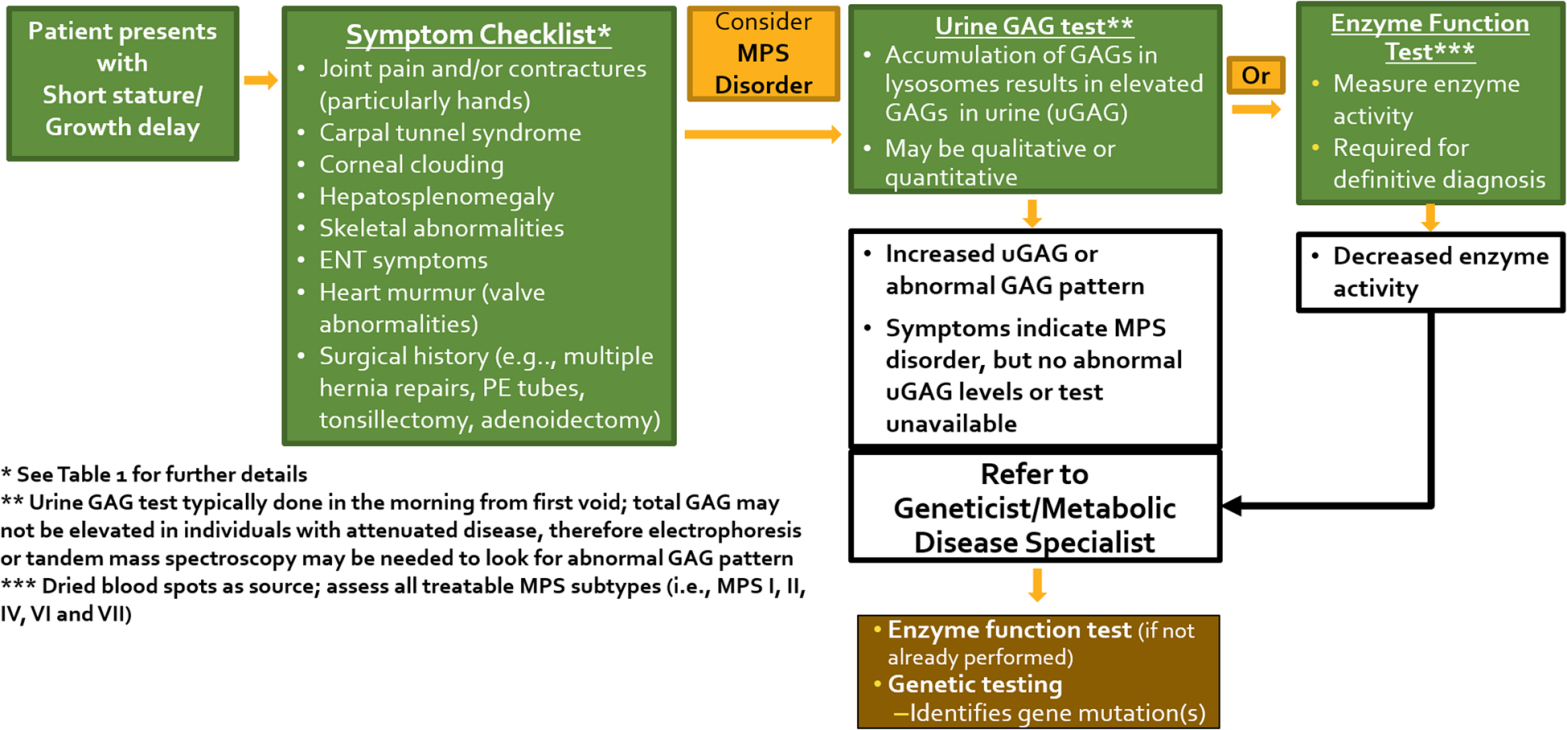
Symptom Checklist and Path to MPS I Diagnosis. The algorithm describes the key symptoms of MPS I and the pathway of diagnostic tests for a patient presenting with short stature or growth delay

The case study demonstrates that endocrinologists may not consider MPS I in cases of short stature, even when there are signs and symptoms suggestive of MPS I. Red-flag signs and symptoms for attenuated MPS I (Table 1) exist in the absence of parameters indicating juvenile idiopathic arthritis [39, 40]. The case presentation highlights the diagnostic journey of a patient with attenuated MPS I and short stature followed for over 10 years in a pediatric endocrinology clinic. A strength of this case is the duration of care and longitudinal growth data, although in some instances, assessments and clinical management information were unavailable. This patient had many of the signs and symptoms of attenuated MPS I, yet was not diagnosed until nearly 11 years after presenting to the pediatric endocrinologist with short stature. While not all of the signs listed in Table 1 may be apparent at the initial patient presentation to the endocrinologist, they are likely to develop over time when untreated, or be documented in patient clinical records and history. It is important to note that assessment of bone age in children with growth delay is typically done with an X-ray of the left hand and wrist; thus, pediatric endocrinologists are ideally situated to identify early phalangeal abnormalities (i.e., bullet shaped phalanges) typical of MPS I. A path to MPS I diagnosis when indicating signs are present is shown in Fig. 3. There is considerable overlap of presenting symptoms among the MPS disorders, therefore, screening identified in Fig. 3 should take into account other MPS disorders where short stature is common. Upon consideration of an MPS disorder, a urine GAG (uGAG) test (that may include analyses to determine abnormal GAG pattern, such as electrophoresis or tandem mass spectrometry) can determine the presence of lysosomal storage material. Results of the uGAG test as indicated in Fig. 3 can warrant referral to a geneticist or metabolic disease specialist, who can initiate appropriate enzyme and genetic testing to confirm or rule out an MPS diagnosis. Several barriers can exist for appropriate referrals of pediatric patients to metabolic specialists and geneticists, including cost and insurance, wait times, and location [41, 42] and improvements in accessibility for lysosomal storage disease assays may be needed [43].

Studies suggest that increased awareness for endocrinologists may be helpful to highlight the possibility of attenuated MPS in patients presenting with short stature [37, 38]. In a retrospective assessment of outpatient medical records of patients with short stature of unknown etiology in a pediatric endocrinology service, follow-up screening of 23 patients revealed previously undiagnosed MPS in 3 patients [37]. In another study, 135 physicians with expertise in pediatrics and endocrinology from seven countries (United States, Canada, Italy, Germany, Spain, Mexico, and Brazil) participated in a blinded review of cases for pediatric or adolescent patients with MPS I [38]. Depending on the case reviewed, only 22% to 58% of physicians took steps towards a correct MPS I diagnosis. Juvenile idiopathic arthritis was the most common incorrect diagnosis made. A key distinction in the diagnosis of MPS I is the absence of biochemical parameters diagnostic of juvenile idiopathic arthritis. While algorithms exist that include MPS I in the differential diagnosis of juvenile arthritis for pediatric rheumatologists [39, 40], growth specialists and endocrinologists may be among the physicians encountering individuals with undiagnosed MPS disorders, and similar guidelines could prove helpful for recognizing the red-flag signs and symptoms of MPS I and other MPS disorders. A proposed algorithm that includes short stature as a presenting sign in attenuated MPS I has recently been published [44].

The mechanism behind short stature in patients with MPS I is not completely known, but is most likely a secondary characteristic resulting from structural, metabolic, and endocrine abnormalities. Structurally, skeletal abnormalities limit longitudinal growth and final height, but alone cannot explain short stature in patients with MPS I. Pituitary and thyroid dysfunction, GHD, precocious puberty, and pubertal failure have all been reported in patients with MPS I [32, 45]. However, it is important to note that in the absence of GHD, hGH treatment of patients with MPS has not been proven to be effective.

Enzyme replacement therapy with laronidase has resulted in increased growth velocity in pediatric patients [46], particularly in prepubescent children with MPS I [20]. In sibling studies, improved musculoskeletal outcomes were noted in the younger sibling who began ERT in infancy [5, 18, 19]. Retrospective studies indicate that early initiation of laronidase can stabilize existing skeletal disease, and prevent or delay clinical manifestations if initiated prior to symptom onset [5, 19–22]. Patients with attenuated MPS I that were less than 10 years of age at treatment initiation remained closer to age-matched norms for several disease parameters, including height, compared with patients that were ≥ 10 years of age at the start of treatment [22]. There is disagreement regarding the benefits of administration of recombinant human growth hormone, and this is an area of active study [47].

In summary, short stature is a common presenting sign of attenuated MPS I, and may be the symptom that drives clinical care in these patients [36, 48, 49]. Since pediatric endocrinologists are typically the first physician to whom patients with short stature are referred [35], they can play a pivotal role in improving the health and quality of life of patients with attenuated MPS I. Early diagnosis of MPS I and initiation of treatment is critically important as it improves patient outcomes and reduces disease burden [5, 6, 8, 19, 22]. MPS I should be considered in any patient with short stature and/or growth failure plus one or more of the common signs described in Table 1. The path to diagnosis (Fig. 2) includes urine GAG test, referral to geneticist (or metabolic disease specialist), and appropriate enzyme and genetic testing. Improving the ability of pediatric endocrinologists to recognize the disease manifestations of MPS I can lead to earlier diagnosis and treatment for individuals with MPS I.

## Abbreviations

GAG: Glycosaminoglycans
GHD: Growth hormone deficient
hGH: Human growth hormone
HSCT: Hematopoeitic stem cell transplant
IDUA: α-L-iduronidase
MPS I: Mucopolysaccharidosis type I
uGAG: Urinary glycosaminoglycans
